# Downregulation of microRNA-199b predicts unfavorable prognosis and emerges as a novel therapeutic target which contributes to PP2A inhibition in metastatic colorectal cancer

**DOI:** 10.18632/oncotarget.11174

**Published:** 2016-08-10

**Authors:** Ion Cristóbal, Cristina Caramés, Raúl Rincón, Rebeca Manso, Juan Madoz-Gúrpide, Blanca Torrejón, Paula González-Alonso, Federico Rojo, Jesús García-Foncillas

**Affiliations:** ^1^ Translational Oncology Division, Oncohealth Institute, IIS-Fundacion Jimenez Diaz, UAM, University Hospital “Fundacion Jimenez Diaz”, E-28040 Madrid, Spain; ^2^ Pathology Department, University Hospital “Fundacion Jimenez Diaz”, Autonomous University of Madrid, E-28040 Madrid, Spain

**Keywords:** miR-199b, SET, PP2A, prognosis, therapy

## Abstract

The tumor suppressor microRNA-199b (miR-199b) is a negative SET regulator associated with poor outcome in some human cancers. However, its expression levels as well as potential biological and clinical significance in colorectal cancer (CRC) remain completely unexplored. The PP2A inhibitor SET has shown promising therapeutic and clinical implications in metastatic CRC (mCRC) but the molecular mechanisms underlying SET deregulation are currently unknown. We show here miR-199b downregulation in 4 out of 5 CRC SET-overexpressing cell lines and its inverse correlation with SET overexpression in CRC patients. Moreover, miR-199b led to PP2A activation through a direct SET inhibition, impaired cell viability and enhanced oxaliplatin sensitivity in CRC cells. MiR-199b was found downregulated in 25% of cases, and associated with lymph metastasis (*p* = 0.049), presence of synchronous metastasis at diagnosis (*p* = 0.026) and SET overexpression (*p* < 0.001). Furthermore, low miR-199b levels determined shorter overall (*p* < 0.001), progression-free survival (*p* = 0.003) and predicted clinical benefit to oxaliplatin treatment. The miR-199b prognostic impact was particularly evident in both younger and *KRAS* wild-type subgroups. Multivariate analyses confirmed its independent prognostic impact. Altogether, our results show that miR-199b is a tumor suppressor whose downregulation independently determines worse outcome and emerges as a potential contributing mechanism to inhibit PP2A via SET overexpression in a subgroup of mCRC patients.

## INTRODUCTION

Colorectal cancer (CRC) represents the third most commonly diagnosed cancer and the fourth highest cause of cancer-related deaths worldwide [1]. Altough the CRC stage at diagnosis is the most predictive factor of clinical outcome, more than 70% of CRC cases newly diagnosed have a surgically resectable localized disease [2]. However, the remaining 20–30% of newly diagnosed CRC patients with unresectable distant metastasis together with a substantial proportion of cases who develop metachronous metastasis represents the subgroup of patients with worst outcome [3]. Therefore, it remains necessary to improve our molecular knowledge of CRC to identify alterations with both prognostic and predictive value of therapy efficacy to develop novel and more efficient targeted therapies.

The protein phosphatase 2A (PP2A) is a well-known tumor suppressor that inhibits signaling pathways critical in human cancer [4, 5]. Several works highlighting the molecular and clinical significance of PP2A inhibition in CRC have been reported [6–9]. The protein SET is a potent endogenous PP2A inhibitor [10] involved in many cell functions [11–15] and a novel proposed target for anticancer therapy [16]. Interestingly, some evidences suggest that SET could be relevant in CRC progression [17]. In fact, our group has recently reported that SET deregulation determines poor outcome and defines a subgroup of metastatic CRC patients who could benefit from therapies containing PP2A activators [18]. MicroRNAs (miRs) are small non-coding RNAs that inhibit specific target genes by translation repression and they then can function as oncogenes or tumor suppresors in human cancer. MiR-199b is a SET inhibitor [19, 20] which has also been involved in acquired chemoresistance in chronic myeloid leukaemia or ovarian cancer [21, 22]. Moreover, miR-199b also functions as a tumor suppressor in medulloblastoma, hepatocellular carcinoma and breast cancers by affecting targets such as HEIS1, HIF1α or HER2 [23–25]. However, its status and potential significance in colorectal cancer is completely unknown.

In this report, we identified miR-199b downregulation as a common alteration with high clinical relevance that represents a potential contributing mechanism to SET overexpression in metastatic CRC patients. Interestingly, low miR-199b levels inversely correlated with SET expression and independently predicted shorter overall and progression-free survival defining a subgroup of metastatic CRC patients with very poor outcome candidate to be treated with SET/PP2A targeting drugs such as FTY720.

## RESULTS

### MiR-199b is downregulated and affects SET expression and PP2A activation status in CRC cells

**Figure 1:**
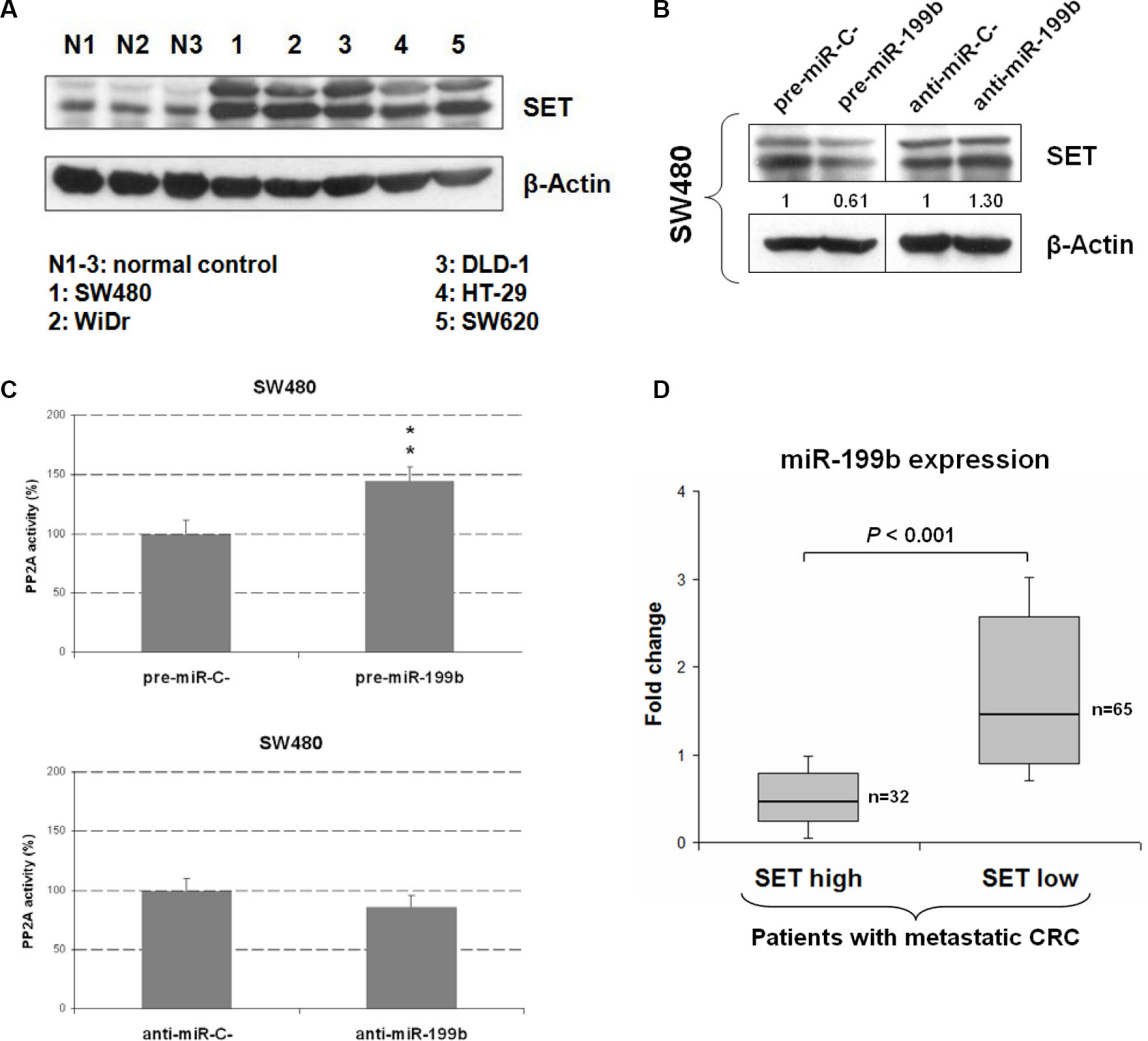
MiR-199b downregulation is a common alteration in mCRC that regulates SET and PP2A activity (**A**) Western blot analysis showing SET expression levels in 5 CRC cell lines. (**B**) Western blot analysis showing SET expression in SW480 cells transfected with pre- or anti-miR-199b; (**C**) PP2A assays showing changes in PP2A activity in SW480 cells after transfection with pre- or anti-miR-199b. Data represented are mean of three independent experiments ± SD. *P < 0.05; **P < 0.01; (**D**) Box-plot showing miR-199b expression levels in patients with (*N* = 35) and without (*N* = 62) SET overexpression; N1-3: normal controls.

We quantified miR-199b in 5 different CRC cell lines, observing low miR-199b levels in 4 out of the 5 CRC cell lines compared to normal colonic mucosa (Supplementary Table S1). Moreover, western blot analysis showed SET overexpression in the same 5 CRC cell lines (Figure 1A). The same normal controls were used in both experiments. We first performed luciferase assays to validate the role of miR-199b as a negative SET regulator in CRC. Transfection of pSET-3′UTRwt in SW480 cells ectopically expressing miR-199b showed decreased luciferase activity, indicating that miR-199b binds to the SET 3′UTR, negatively regulating its expression. Analysis using the same construct with the mutated miR-199b seed region showed no changes in luciferase activity, confirming that miR-199b directly binds to SET (Supplementary Figure S1). We next assessed the effects of miR-199b modulation on SET expression in SW480 cells using pre- and anti-microRNAs specific for miR-199b. As expected, we found decreased and increased SET levels in SW480 cells transfected with pre- and anti-miR-199b, respectively (Figure 1B). Similar results were obtained using HT-29 cells (Supplementary Figure S2A).

Due to SET is an endogenous PP2A inhibitor and miR-199b negatively regulates SET, we analyzed whether miR-199b deregulation could modulate PP2A in CRC cells. As expected, we observed PP2A activation in both SW480 and HT-29 cells after pre-miR-199b transfection. Although transfection with anti-miR-199b induced PP2A inhibition significance was only achieved in HT-29 cells. (Figure 1C and Supplementary Figure S2B). These results prompted us to analyze SET and miR-199b expression levels in a cohort of 97 patients with metastatic CRC. Patient characteristics are presented in Supplementary Table S2. Interestingly, a negative correlation was found between miR-199b and SET expression (Supplementary Figure S3). Moreover, significant lower miR-199b was significantly downregulated in the subgroup of patients with SET overexpression (Figure 1D), suggesting that altered expression miR-199b is a molecular mechanism that contributes to deregulate SET and PP2A activation status in CRC patients.

### MiR-199b impairs cell viability in a SET-dependent manner

**Figure 2:**
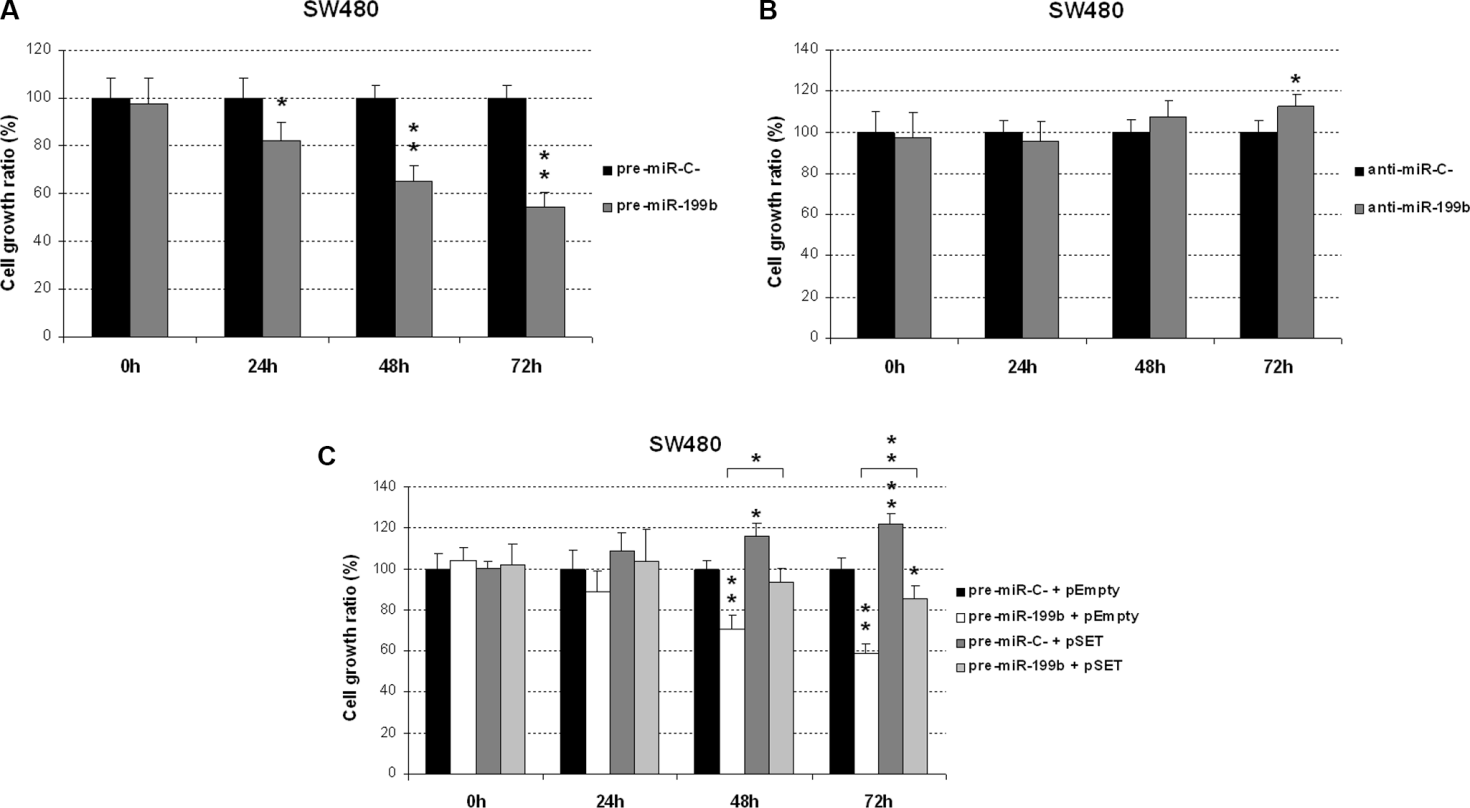
MiR-199b impairs cell proliferation in CRC cells MTS assay showing proliferation in SW480 cells transfected with pre-miR-199b (**A**), anti-miR-199b (**B**) or both SET and pre-miR-199b (C). Data represented are mean of three independent experiments ± SD. *P < 0.05; **P < 0.01.

To investigate its biological relevance as a potential tumor suppressor in CRC, we assessed the effects of miR-199b modulation on cell growth. Interestingly, we observed a reduced proliferation in SW480 cells transfected with a pre-miR-199b in comparison with those transfected with a negative control (Figure 2A). These results were confirmed with the HT-29 cell line (Supplementary Figure S4A). However, only slight effects on cell growth were found by anti-miR-199b in SW480 and HT-29 cells (Figure 2B and Supplementary Figure S4B). Of importance, we also observed that ectopic expression of SET significantly restored cell proliferation in SW480 cells transfected with pre-miR-199b (Figure 2C). Similar results were found in HT-29 cells (Supplementary Figure S4C). Altogether, these results would indicate that SET regulation is a key event which mediates miR-199b-induced antitumor effects in CRC.

### MiR-199b sensitizes CRC cells to oxaliplatin and 5-FU treatments

**Figure 3:**
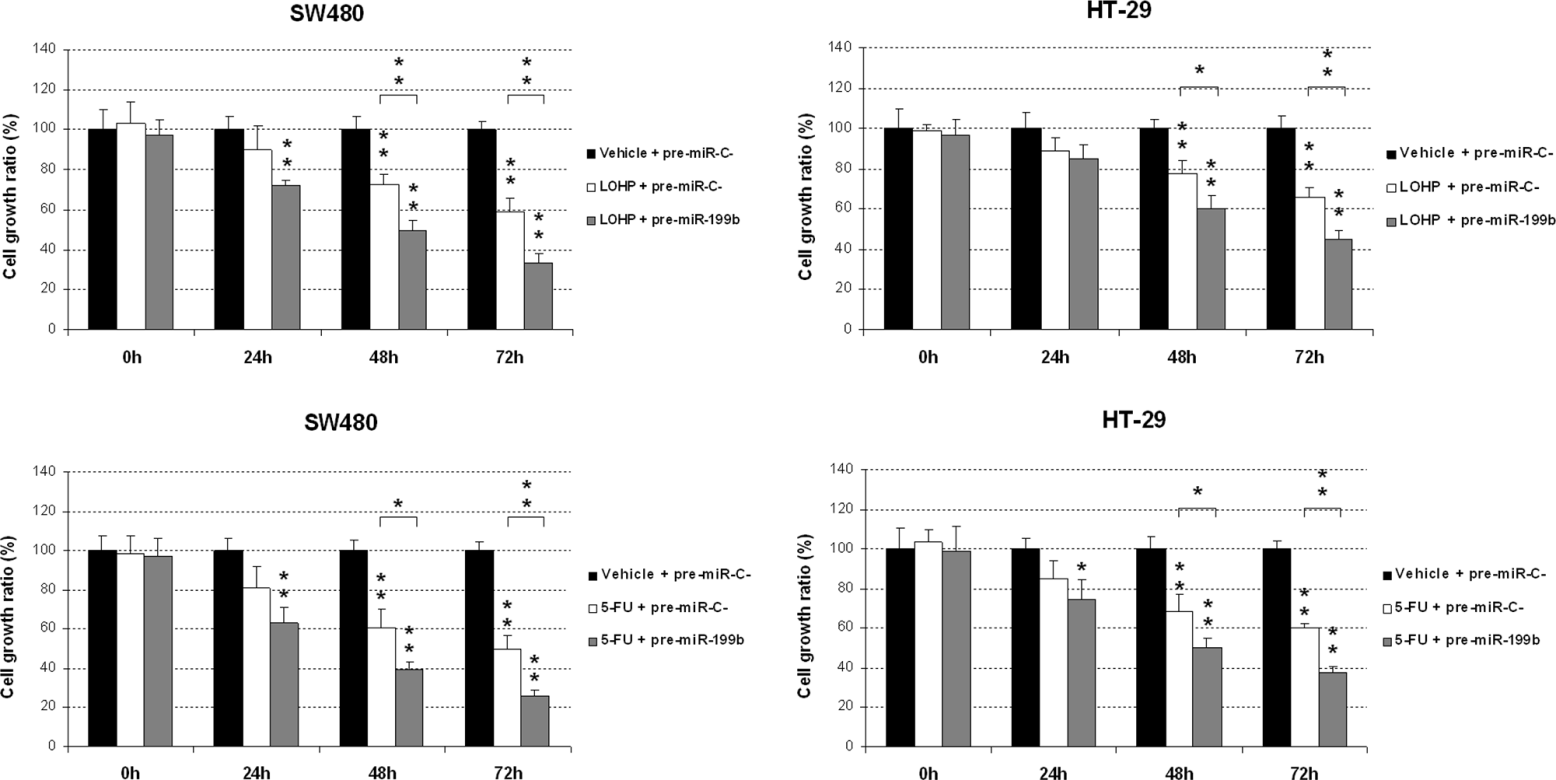
MiR-199b sensitizes CRC cells to oxaliplatin and 5-FU treatments MTS assays showing the effect of miR-199b in the sensitivity to oxaliplatin (**A**) or 5-FU (**B**) in SW480 and HT-29 cells; Data represented are mean of three independent experiments ± SD. *P < 0.05; **P < 0.01.

We next investigated the potential therapeutic role of miR-199b affecting sensitivity of CRC cells to standard chemotherapy drugs such as oxaliplatin and 5-FU. Interestingly, we found that miR-199b-overexpressing SW480 cells showed higher sensitivity to oxaliplatin treatment. These results were confirmed in the HT-29 cell line (Figure 3A). Similarly, we observed an enhanced sensitivity to 5-FU treatment in both SW480 and HT-29 cells transfected with pre-miR-199b (Figure 3B). In order to assess whether miR-199b affects oxaliplatin sensitivity through SET inhibition, we modulated SET expression in oxaliplatin treated SW480 and HT-29 cells ectopically expressing miR-199b. Interestingly, we observed that SET overexpression was able to restore oxaliplatin sensitivity (Supplementary Figure S5), suggesting that miR-199b regulates oxaliplatin sensitivity in CRC cells through a SET negative regulation.

### Prevalence of miR-199b downregulation in metastatic colorectal cancer and its association with molecular and clinical parameters

In order to investigate whether miR-199b is deregulated in CRC patients, we analyzed miR-199b expression levels in a cohort of 97 patients with metastatic CRC. Mir-199b was found downregulated in 24 of 97 cases (24.7%). Patient characteristics are presented in Table S2. Interestingly, we found low miR-199b expression associated with development of lymph metastasis (37.9% versus 19.1%, *p* = 0.049), presence of synchronous metastasis at diagnosis (32.8% versus 12.8%, *p* = 0.026) and SET overexpression (53.1% versus 10.8%, *p* < 0.001). Association between miR-199b downregulation and molecular and clinical parameters are included in Table 1. Interestingly, we observed miR-199b dowregulated in 17 out of 32 cases with SET overexpression, suggesting that low miR-199 is a relevant contributing alteration to deregulate SET in a subgroup of CRC patients. Of importance, these findings would also indicate the existence of alternative mechanisms in those SET-overexpressing cases without miR-199b downregulation that should have to be elucidated in future studies.

**Table 1:** Association between miR-199b and clinical and molecular parameters in 97 patients with metastatic CRC

	No. Cases	High miR-199b (%)	Low miR-199b (%)	*P*
miR-199b	97	73 (75.3)	24 (24.7)	
Sex	97	73	24	0.830
Male	67	50 (74.6)	17 (25.4)	
Female	30	23 (76.7)	7 (23.3)	
Age	93	71	22	0.079
< 70	44	30 (68.2)	14 (31.8)	
≥ 70	49	41 (83.7)	8 (16.3)	
ECOG	92	71	21	0.175
0–2	75	60 (80)	15 (20)	
3–4	17	11 (64.7)	6 (35.3)	
Site of primary tumor	97	73	24	0.524
Colon	72	53 (73.6)	19 (26.4)	
Rectum	25	20 (80)	5 (20)	
Synchronous metastasis	97	73	24	**0.026**
No	39	34 (87.2)	5 (12.8)	
Yes	58	39 (67.2)	19 (32.8)	
Number of metastatic sites	97	73	24	0.383
1–2	89	68 (76.4)	21 (23.6)	
> 2	8	5 (62.5)	3 (37.5)	
Liver metastasis	97	73	24	0.282
No	33	27 (81.8)	6 (18.2)	
Yes	64	46 (71.9)	18 (28.1)	
Lung metastasis	97	73	24	0.264
No	68	49 (84.5)	19 (15.5)	
Yes	29	24 (82.8)	5 (17.2)	
Lymph metastasis	97	73	24	**0.049**
No	68	55 (80.9)	13 (19.1)	
Yes	29	18 (62.1)	11 (37.9)	
Peritoneal metastasis	97	73	24	0.173
No	78	61 (78.2)	17 (21.8)	
Yes	19	12 (63.2)	7 (36.8)	
MSI	95	71	24	0.617
No	89	66 (74.2)	23 (25.8)	
Yes	6	5 (83.3)	1 (16.7)	
*KRAS* mutated	97	73	24	0.755
No	58	43 (74.1)	15 (25.9)	
Yes	39	30 (76.9)	9 (23.1)	
SET overexpression	97	73	24	**< 0.001**
No	65	58 (89.2)	7 (10.8)	
Yes	32	15 (46.9)	17(53.1)	

### Clinical significance of miR-199b downregulation in metastatic colorectal cancer

**Figure 4:**
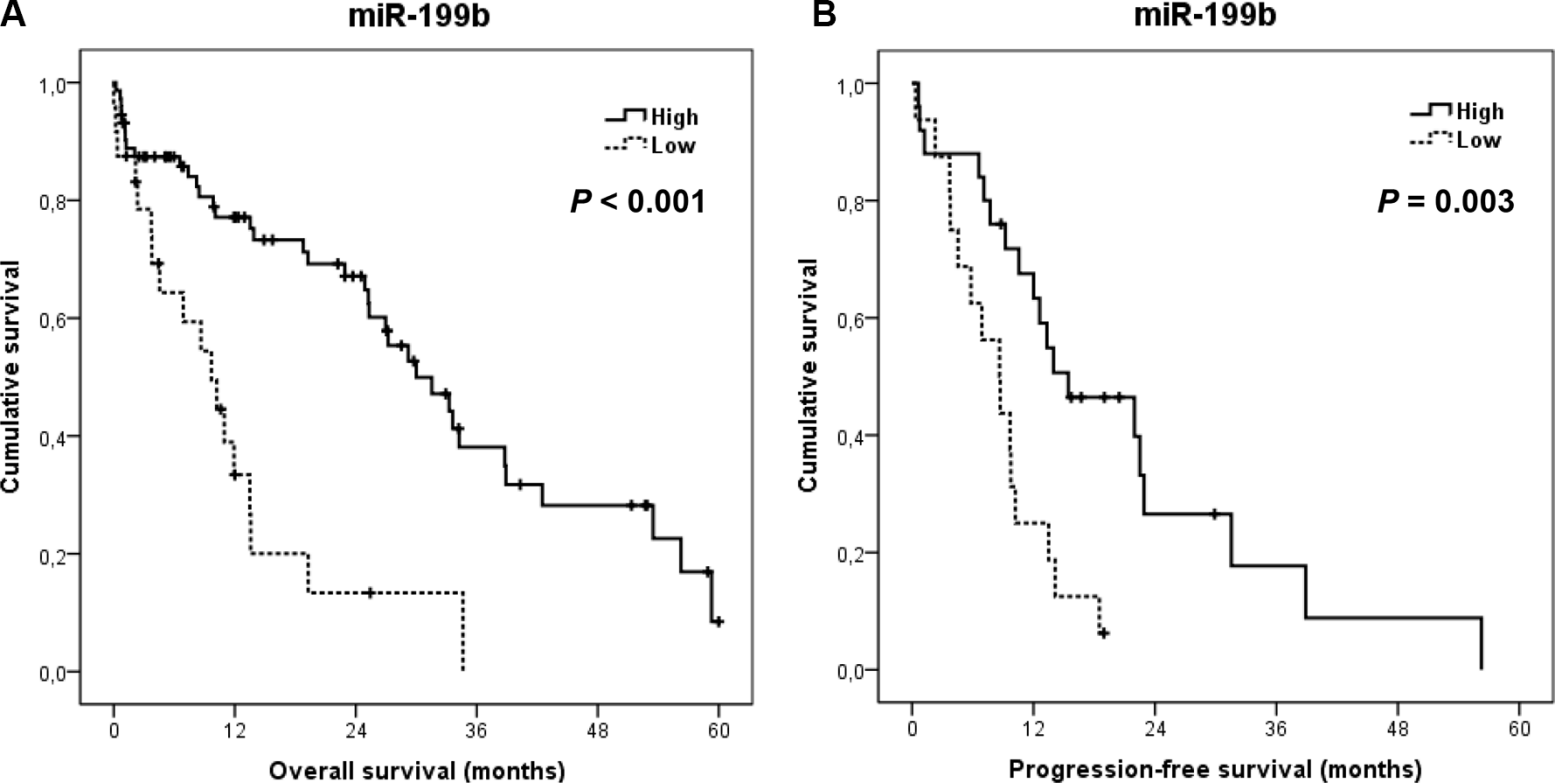
Clinical significance of miR-199b expression levels in metastatic CRC Kaplan-Meier analyses of overall survival (**A**) and progression-free survival (**B**) in a cohort of 97 metastatic CRC patients.

We next investigated the potential clinical significance of miR-199b in mCRC. Clinical follow-up data were available for all the 97 patients included in the study, 67 male and 30 female, with a median of age of 70 years (range: 40–89). The median OS of the global cohort was 25.3 months (95% confidence interval (CI): 16.2–34.4). Of relevance, we found that those patients with low miR-199b expression showed a substantially shorter OS (median OS, 9.7 versus 30 months, *p <* 0.001) (Figure 4A) and PFS (median PFS, 8.6 versus 15.4 months, *p* = 0.003) (Figure 4B).

**Figure 5:**
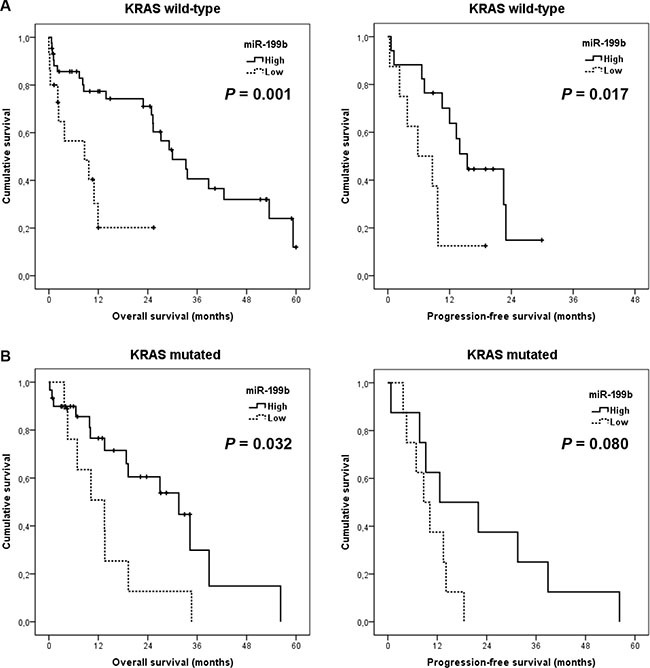
Prognostic impact of miR-199b in metastatic CRC patients stratified by *KRAS* mutation status Kaplan-Meier analyses of overall survival and progression-free survival in *KRAS* wild type (*N* = 58) (**A**) and *KRAS* mutated subgroups (*N* = 39) (**B**).

We next stratified our cohort by *KRAS* mutation status, observing that miR-199b shows higher prognostic value in those patients with wild-type *KRAS* (median OS, 8.6 versus 30 months, *p* = 0.001; median PFS, 5.8 versus 15.4 months, *p* = 0.017) than in those cases with mutated *KRAS* (median OS, 13.5 versus 31.5 months, *p* = 0.032; median PFS, 8.7 versus 12.6 months, *p* = 0.080) (Figure 5). Moreover, miR-199b had significant prognostic value in OS in both subgroups of patients younger (median OS, 11.9 versus 34.2 months, *p* = 0.003) and older than 70 years (median OS, 3.9 versus 26.9 months, *p* = 0.002). Although miR-199b predicted PFS in younger patients (median PFS, 9.7 versus 22.5 months, *p* = 0.009), significance in PFS was not achieved in the subgroup of elderly cases (median PFS, 3.8 versus 12 months, *p* = 0.119) (Supplementary Figure S6). Importantly, we observed that miR-199b downregulation was predictive of clinical benefit in those patients who received oxaliplatin-based chemotherapy (*N* = 39; *p* = 0.018) (Table S3). Of relevance, multivariate analysis demonstrated that ECOG and miR-199b downregulation have an independent prognostic value in our patient cohort in both OS (Table 2) and PFS (Supplementary Table S4). Additionally, we also analyzed the clinical significance of the PP2A inhibitor SET in our series. Immunohistochemical detection of SET is shown in Supplementary Figure S4. As expected, we confirmed that those patients with SET overexpressed showed a substantially shorter OS (median OS, 9.9 versus 31.5 months, *p <* 0.001) and PFS (median PFS, 8.7 versus 18.5 months, *p* = 0.009) (Supplementary Figure S7).

**Table 2:** Univariate and multivariate Cox analyses in the cohort of 97 patients with mCRC

	Univariate OS analysis				Multivariate OS Cox analysis			
	95% CI				95% CI			
	HR	Lower	Upper	Significance	HR	Lower	Upper	Significance
Age				0.363				–
< 70	1.00							
≥ 70	1.29	0.74 to 2.25			–	–		
Gender				0.227				–
Male	1.00							
Female	0.69	0.38 to 1.25			–	–		
Synchronous				0.096				
No	1.00							
Yes	1.66	0.91 to 3.02			–	–		
ECOG				**< 0.001**				**< 0.001**
0–2	1.00				1.00			
3–4	2.04	1.46 to 2.84			1.86	1.32 to 2.62		
Number of metastatic sites				0.589				–
1–2	1.00							
> 2	1.13	0.71 to 1.80			–	–		
MiR-199b downregulation				**< 0.001**				**0.003**
No	1.00				1.00			
Yes	3.46	1.88 to 6.38			2.72	1.41 to 5.24		

Furthermore, we analyzed miR-199b levels in primary and paired liver metastatic tissues from 10 CRC patients in order to investigate the potential significance of miR-199b in CRC cell metastasis development. We quantified miR-199b levels using Taqman Low Density Arrays (TLDAs) panel A (Applied Biosystems). Interestingly, we found lower miR-199b levels in liver metastatic tissues compared to their paired primary CRC tissues (*p* = 0.047) (Supplementary Figure S8). Altogether, these preliminary results suggest that miR-199b overexpression could be playing a role in CRC liver metastasis development. Finally, we analyzed the potential role of miR-199b deregulation on *CD133* expression. We generated colonosphere-derived cells from the DLD-1, SW480 and HT-29 cell lines in which we observed *CD133* enrichment together with miR-199b downregulation (Supplementary Figure S9A). Although 3-fold increase in *CD133* expression was observed in DLD-1 colonospheres no expression of miR-199b was detected, similarly than in DLD-1 parental cells (data not shown). In addition, CD133 expression could be quantified in 64 CRC patients from our cohort with enough material available. Interestingly, we found that miR-199b and *CD133* expression show a negative correlation (Supplementary Figure S9B), further suggesting that miR-199b deregulation could be affecting the *CD133* expression status of CRC cells.

## DISCUSSION

Our group has recently reported that SET overexpression is a marker of poor outcome in metastatic CRC patients which defines a subgroup of patients candidate to be treated with PP2A activating drugs [18]. However, how SET deregulation occurs in CRC remains fully unknown. We then evaluated molecular causes that could lead to SET overexpression in CRC, analyzing whether an altered expression of miR-199b could be involved in SET deregulation in CRC. Thus, we first quantified miR-199b in 5 CRC cell lines previously reported to have SET overexpression [6], observing that miR-199b was downregulated in 4 out of 5 cases. This observation together with the significant lower miR-199b expression found in the subgroup of SET-overexpressing mCRC patients suggest a role of miR-199b in SET-mediated PP2A inhibition in CRC. This issue was further supported by the decrease in SET levels together with PP2A activation observed after miR-199b overexpression in CRC cell lines. Of note, the transfection with anti-miR-199b only promoted a slight reduction in PP2A activity probably due to the low basal miR-199b expression together with the SET overexpression status in both SW480 and HT-29 cells. These findings are in concordance with the fact that anti-miR-199b only induced a discrete increase of cell viability whereas miR-199b overexpression led to a marked reduction of cell growth. Moreover, the antitumor effects of miR-199b on cell growth is probably due to its role as negative SET regulator since the co-expression of miR-199b together with SET almost totally restored proliferation of CRC cells. Thus, miR-199b emerges as a novel tumor suppressor in CRC and its downregulation is a common alteration which contributes to PP2A inhibition in this disease.

Furthermore, miR-199b has been reported to be involved in acquired resistance to different antitumor therapies in human cancer such as imatinib in chronic myeloid leukemia [21], cisplatin in ovarian cancer [22] or trastuzumab in breast cancer [25]. Thus, we evaluated whether miR-199b could affect we observed that miR-199 sensitize CRC cells to both oxaliplatin and 5-FU treatments. These findings are concordant with the fact that miR-199b negatively regulates SET, which has been described to modulate resistance to oxaliplatin and 5-FU treatments in CRC [18].

Despite some data in the literature describe miR-199b tumor suppressor roles in human cancer [20–25], nothing is known about its function in CRC. As indicated above, miR-199b has been reported to have prognostic value in hepatocellular and papillary thyroid carcinomas [24, 26]. Therefore and considering that miR-199b seems to be a molecular cause of SET overexpression in a subgroup of metastatic CRC patients, we hypothesized that miR-199b downregulation could have clinical impact in metastatic CRC. Of importance, miR-199b downregulation determined poor outcome and clinical benefit in those cases treated with oxaliplatin-based chemotherapy. This observation is in concordance with our *in vitro* results and further supports that miR-199b increases sensitivity to oxaliplatin in CRC cells.

The *KRAS* mutation status is a key molecular factor in determining clinical benefit to cetuximab in CRC [27]. Therefore, we evaluated the clinical impact of miR-199b stratifying our cohort by the presence or not of *KRAS* mutations. Thus, we found that miR-199b downregulation showed higher prognostic impact in both OS and PFS in the KRAS wild type subgroup. These findings could have a potential therapeutic relevance since FTY720, a PP2A-activating drug that binds and blocks SET [28], has recently shown to resensitize CRC cell to cetuximab [29] and our results suggest that miR-199b could be playing a role via SET regulation.

In addition to SET, miR-199b has been reported to regulate other important targets such as HEIS1, HIF1α or HER2 in medulloblastoma, hepatocellular carcinoma and breast cancers [23–25]. Among those metastatic CRC patients without SET overexpression, we observed miR-199b downregulated in 7 out of 65 cases. Of importance, we observed that miR-199b downregulation determined substantially shorter OS in these patients (median OS, 11 versus 31.5 months, *p* = 0.052), although significance was not achieved probably by the low number of cases studied. Therefore, these observations would indicate a potential SET-independent prognostic value for miR-199b which needs to be further confirmed in forthcoming studies. Moreover, multivariate analyses demonstrated that miR-199b downregulation was an unfavorable independent factor associated with OS and PFS in mCRC, which further confirm its prognostic value in this disease.

In concordance with previous observations in medulloblastoma [23], we show lower miR-199b expression in CRC with metastatic disease. In their work, Garzia et al. provided relevant findings supporting that miR-199b downregulation in metastatic medulloblastoma cells was probably due to a methylation-based epigenetic regulation of this microRNA. Therefore, it remains necessary to evaluate in future investigations whether a similar mechanism of transcriptional regulation is also occurring in CRC cells. On the other hand, the Notch signaling pathway plays a relevant role in self-renewing processes and its inhibition has been described to decrease CD133+ tumor cells [23, 30]. Of importance, a negative feedback loop of regulation has been reported between miR-199b and HES1, a key Notch effector, then impairing the CD133+ stem cell-like subpopulation of tumor cells [23, 31]. Interestingly, we show here that miR-199b is downregulated after colonosphere generation, which are CD133-enriched cells. Moreover, we analyzed *CD133* in 64 metastatic CRC patients observing a negative correlation between *CD133* and miR-199b. These results would indicate a potential relationship between miR-199b and CD133 in CRC cells that needs to be further explored in forthcoming studies.

In conclusion, our results show that miR-199 downregulation is a frequent alteration in metastatic CRC that emerges as a novel therapeutic target and a contributing mechanism to SET overexpression in this disease. Interestingly, our findings indicate that miR-199 downregulation is a common event that plays an oncogenic role in CRC cells. Moreover, this alteration has an independent prognostic value predicting poor outcome in metastatic CRC patients and could have important therapeutic implications via SET-dependent PP2A inhibition in metastatic CRC.

## MATERIALS AND METHODS

### Cell cultures and transfection

The human CRC cell lines SW480 (ATCC CCL-228), WiDr (ATCC CCL-218), DLD-1 (ATCC CCL-221), HT-29 (ATCC HTB-38) and SW620 (ATCC CCL-227) were purchased from American Type Culture Collection (ATCC). Authentication was done by the authors in all cases (LGC Standards). Cell lines were maintained in RPMI-1640 (Invitrogen) with 10% fetal bovine serum and were grown at 37°C in a 5% CO2 atmosphere. Media were supplemented with penicillin G (100 U/ml), and streptomycin (0.1 mg/ml). Cells were treated with oxaliplatin (LOHP) (1 μM) (Sigma), 5-fluorouracil (5-FU) (1 μM) (Sigma) and FTY720 (10 μM) (Calbiochem) as previously reported [6, 9]. For transfection experiments, CRC cells were seeded in 6-well plates and transfected with 10 μl of Lipofectamine 2000 (Life Technologies) and 2 μg of SET plasmidic vector or 20 nM of a miR-199b specific *mir*Vana™ miRNA Mimic and Inhibitor (Ambion).

### Patient samples

Primary colorectal tissues were surgical resection specimens from CRC tumors obtained from Fundacion Jimenez Diaz Biobank (BFJD, Madrid). The study comprised consecutive FFPE tumor samples of 97 patients with metastatic CRC that were retrospectively selected from 2001 to 2012 according to the following criteria: adenocarcinoma, operable disease, no neoadjuvant therapy, enough available tissue, clinical follow-up data and metastatic disease. TNM (Tumor, Node, Metastases) staging was classified using the 7th American Joint Committee on Cancer (AJCC) staging system for colorectal cancer. Clinical data were collected from medical clinical records by oncologists. KRAS mutational status was determined by Cobas KRAS Mutation Test kit (Roche Molecular Diagnostics) following manufacturer's procedures. Tissue microarrays (TMA) were constructed. Representative areas of each tumor were carefully selected and three tissue cores (1 mm diameter) were obtained using a TMA workstation (T1000 Chemicon). Samples were taken anonymously. The ethical committee and institutional review board approved the project.

### Western blot analysis

Protein extracts were isolated using TRIzol Reagent (Invitrogen) following manufacturer´s indications, clarified (12,000 × g, 15 min, 4°C), denatured and subjected to SDS-PAGE and Western-blot. Antibodies used were rabbit polyclonal anti-SET (Abcam) and mouse monoclonal anti-βactin (Sigma). Proteins were detected with the appropriate secondary antibodies conjugated to alkaline phospatase (Sigma) by chemiluminescence using Tropix CSPD and Tropix Nitro Block II (Applied Biosystems).

### Cell viability assay

Cell proliferation was measured in triplicate wells by MTS assay in 96-well plates using the CellTiter 96 AQueous One Solution Cell Proliferation Assay (Promega), following the manufacturer´s indications.

### PP2A phosphatase activity assay

PP2A assays were performed with cell lysates (50 μg) using a PP2A immunoprecipitation phosphatase assay kit (Millipore) as previously described [6].

### Immunohistochemistry

Tissue sections (3 _μ_m) were placed on plus charged glass slides. After deparaffinization in xylene and graded alcohols, heat antigen retrieval was performed in pH9 EDTA-based buffer (Dako). Endogenous peroxidase was blocked by 0.03% hydrogen peroxide for 5 min. Slides were incubated with same primary antibody against SET as described for 60 minutes at room temperature, followed of appropriate anti-Ig horseradish peroxidase-conjugated polymer (Flex+, Dako). Sections were visualized with 3,3′-diaminobenzidine as a chromogen. All stainings were performed in a Dako Autostainer. Sections incubated with normal non-immunized rabbit immunoglobulins were used as negative controls. As positive control, a section of colorectal tumor with known expression of SET was used. SET antibody sensitivity (1:5000) had been calculated in a range of crescent dilutions of primary antibody. Specificity was confirmed in a set of paired fresh frozen and FFPE samples were processed by western blot and IHC. Only the membrane of epithelial cells, but not stromal cells, was evaluated for SET expression blinded to clinical data by two pathologists. A semiquantitative histoscore was calculated by estimation of the percentage of tumor cells positively stained with low, medium, or high staining intensity. The final score was determined after applying a weighting factor to each estimate. The following formula was used: histoscore = (low %) × 1 + (medium %) × 2 + (high %) × 3 and the results ranged from 0 to 300.

### Quantification of miRNA expression levels

Total RNA was isolated using RecoverAll Total Nucleic Acid Isolation kit (Ambion) according to manufacturer's instructions. Samples were reverse transcribed using the TaqManHMicroRNA Reverse Transcription Kit (Applied Biosystems) and mature miRNAs were quantified by quantitative real-time RT-PCR using TaqMan MicroRNA Assays (Applied Biosystems) specific for miR-199b (miR-199b-5p_000500) and U6B as internal control. Analysis of relative gene expression data was performed using the 2^−ΔΔC^T method [32]. The mean expression value of the global cohort (ΔCtcohort) was used to obtain the relative expression of each sample (ΔΔCt) and the fold change calculated as 2^−ΔΔC^T. Downregulation of miR-199b was considered when the expression in a sample was lower than mean minus SD of the patient cohort, corresponding to 0.378 fold change.

### Luciferase assays

Luciferase assays were done using the Dual-Glo Luciferase Assay System (Promega) following the manufacturer´s intructions. SW480 cells were transfected with 20 nM of pre-miR-199b (Ambion) and a pmiR-Glo construct empty or including the SET 3´UTR with the wild type or mutated miR-199b seed region. Firefly luciferase activities were normalized to Renilla luciferase activities.

### Colonospheres

We generated colonosphere-derived cells from DLD-1, SW480 and HT-29 cells using 6-well ultra-low attachment plates (Corning) and 10,000 cells per well. Cells were grown in serum-free DMEM/F12 supplemented with GlutMAX™-I (Gibco) 1% N2 (Gibco), 2% B27 (Gibco), 20 ng/ml human FGF (Sigma) and 50 ng/ml EGF (Sigma). After 7 days, plates were analyzed for colonosphere formation.

### Statistical analysis

Statistical analyses were performed using SPSS 20 for windows (SPSS Inc, Chicago Illinois). Overall survival (OS) was defined as the time from the date of surgery to the date of death from any cause or last follow-up. Progression-free survival (PFS) was defined as the time from surgery until any primary, regional or distant recurrence, appearance of a secondary tumor or death. Kaplan-Meier method and survival comparisons were done with the log-rank test if proportional hazard assumption was fulfilled and Breslow otherwise. The Cox proportional hazards model was adjusted taking into consideration significant parameters in univariate analysis. A *P-value* less than 0.05 was considered statistically significant. Receiver operating curve (ROC) was used to determine the optimal cutoff point based on progression end point for SET expression as previously described method to calculate threshold values for biomarker analysis [33–35]. Following this criteria, high SET exprssion was considered when Hscore in tumor cells were equal or higher than 100. This work was carried out in accordance with Reporting Recommendations for Tumor Marker Prognostic Studies (REMARK) guidelines [36].

## SUPPLEMENTARY MATERIALS FIGURES AND TABLES


