# Supplementary material for: Downregulation of microRNA-199b predicts unfavorable prognosis and emerges as a novel therapeutic target which contributes to PP2A inhibition in metastatic colorectal cancer
Source: Oncotarget. 2016 Aug 10;8(25):40169–80. doi: 10.18632/oncotarget.11174 (PMC5522310; doi:10.18632/oncotarget.11174)
Supplement: Supplementary file 1 [file oncotarget-08-40169-s001.pdf]

Downregulation of microRNA-199b predicts unfavorable prognosis and emerges as a novel therapeutic target which contributes to PP2A inhibition in metastatic colorectal cancer

Supplementary Materials

Supplementary Table S1: Quantification of the expression levels of hsa-mir-199b by real-time PCR in 5 CRC cell lines

Cell line	SET OE	Mir-199b ($-\Delta\Delta C_T$)
SW480	yes	−4,89
WiDr	yes	No expression
DLD-1	yes	No expression
HT-29	yes	−4,30
SW620	yes	−0,25

OE: Overexpression.
$\Delta\Delta C_T = (C_{T,miR\text{-}199b} - C_{T,U6B})_{Cell\ Line} - (C_{T,Target\ Gene} - _{CT,U6B})$Normal Controls. The mean mir-199b expression from a set of 8 normal colonic mucosa samples was used as normal control in this experiment.

Supplementary Table S2: Clinical and molecular characteristics of a series of 97 patients with metastatic CRC

	No.	(%)
Sex		
Male	67	(69.1)
Female	30	(30.9)
Age		
< 70	44	(47.3)
≥ 70	49	(52.7)
No data	4	
ECOG		
0–2	75	(81.5)
3–4	17	(18.5)
No data	5	
MSI		
No	89	(93.7)
Yes	6	(6.3)
No data	2	
KRAS mutations		
No	58	(59.8)
Yes	39	(40.2)
Site of primary tumor		
Colon	72	(74.2)
Rectum	25	(25.8)
Synchronous metastasis		
No	39	(40.2)
Yes	58	(59.8)
Number of metastatic sites		
1–2	89	(91.8)
> 2	8	(8.2)
Liver metastasis		
No	33	(34)
Yes	64	(66)
Lung metastasis		
No	68	(70.1)
Yes	29	(29.9)
Lymph metastasis*		
No	68	(70.1)
Yes	29	(29.9)
Peritoneal metastasis		
No	78	(80.4)
Yes	19	(19.6)
Prior adjuvant chemotherapy**		
No	19	(48.7)
Yes	20	(51.3)
Treatment 1st line metastatic		
Oxaliplatin	39	(41.5)
Irinotecan	13	(13.8)
5-FU	9	(9.6)
None	33	(35.1)
No data	3	

*Non-regional lymph node involvement; **Cases with metachronous metastasis only.

Supplementary Table S3: Predictive value of response to treatment by miR-199b deregulation in those patients who received oxaliplatin-based chemotherapy

	Response			
	Total	No progression (%)	Progression (%)	*p*
Low miR-199b	39	14	25	**0.018**
No	31	14 (100)	17 (68)	
Yes	8	0 (0)	8 (32)	

The Fisher exact test was applied to calculate the *p*-value.

Suppmentary Table S4: Univariate and multivariate Cox analyses in the cohort of 97 patients with metastatic CRC

		Univariate PFS analysis				Multivariate PFS Cox analysis			
			95% CI				95% CI		
		HR	Lower	Upper	Significance	HR	Lower	Upper	Significance
Age					0.102				–
	< 70	1.00							
	≥ 70	1.79	0.89 to 3.62			–	–		
Gender					0.352				–
	Male	1.00							
	Female	0.71	0.34 to 1.45			–	–		
Synchronous					0.480				–
	No	1.00							
	Yes	1.32	0.61 to 2.86			–	–		
ECOG					**0.008**				**0.040**
	0–2	1.00				1.00			
	3–4	1.72	1.15 to 2.57			1.54	1.01 to 2.34		
Number of metastatic sites					0.225				–
	1–2	1.00							
	> 2	1.36	0.82 to 2.26			–	–		
MiR-199b downregulation					**0.005**				**0.037**
	No	1.00				1.00			
	Yes	2.94	1.38 to 6.27			2.32	1.05 to 5.13		

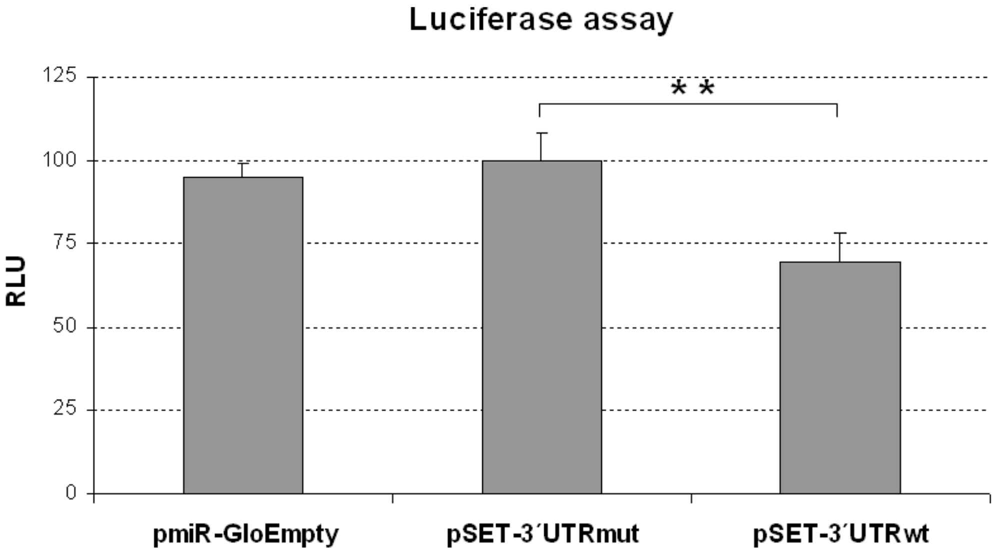

Supplementary Figure S1: Luciferase assay showing changes in Firefly luciferase activity in SW480 cells ectopically expressing miR-199b and transfected with pmiR-Glo empty (negative control), or a pmiR-Glo vector with the 3′UTR region of SET that includes the miR-199b seed region (pSET-3′UTRwt). Transfection with the 3′UTR region of SET including a mutated seed region for miR-199b (pSET-3′UTRmut) was used as reference control. Results were normalized to Renilla luciferase activity and represented as relative luminescence units (RLU). Data represented are mean of three independent experiments ± SD. $^{**}P < 0.01$.

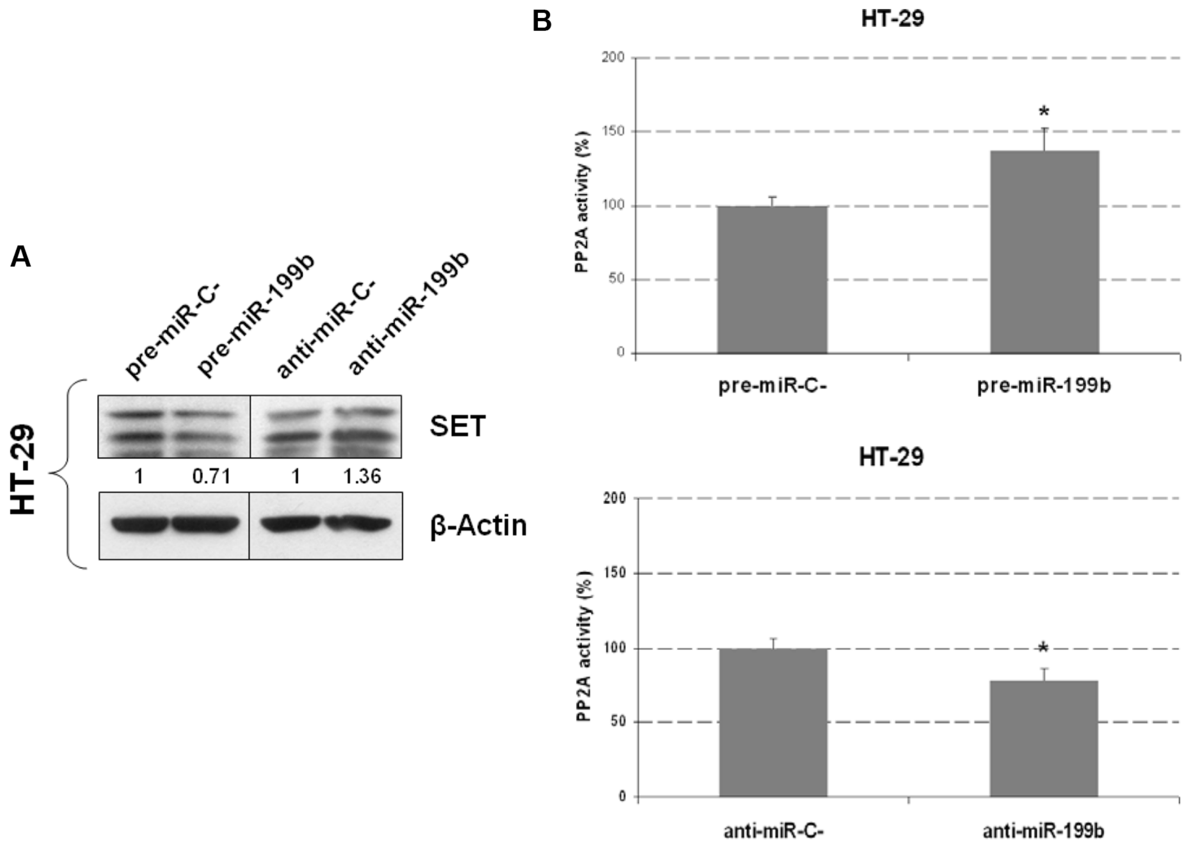

Supplementary Figure S2: (A) Western blot analysis showing SET expression in HT-29 cells transfected with pre- or anti-miR-199b; (B) PP2A assay showing changes in PP2A activity after transfection with pre- or anti-miR-199b.

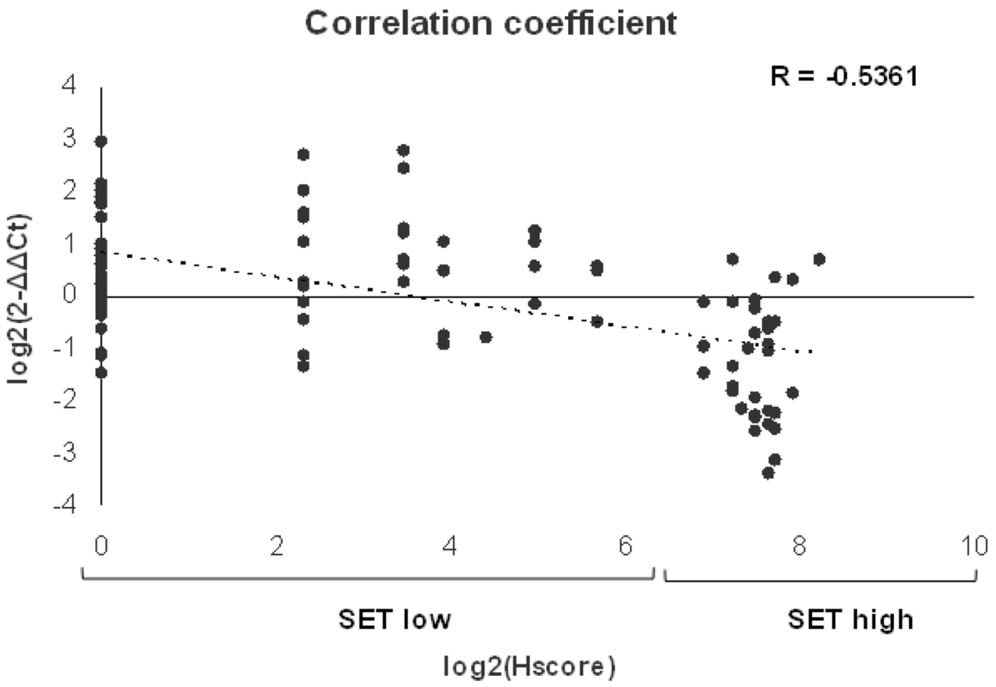

Supplementary Figure S3: Scatter plot showing the correlation between miR-199b and SET expression in 97 CRC patients.

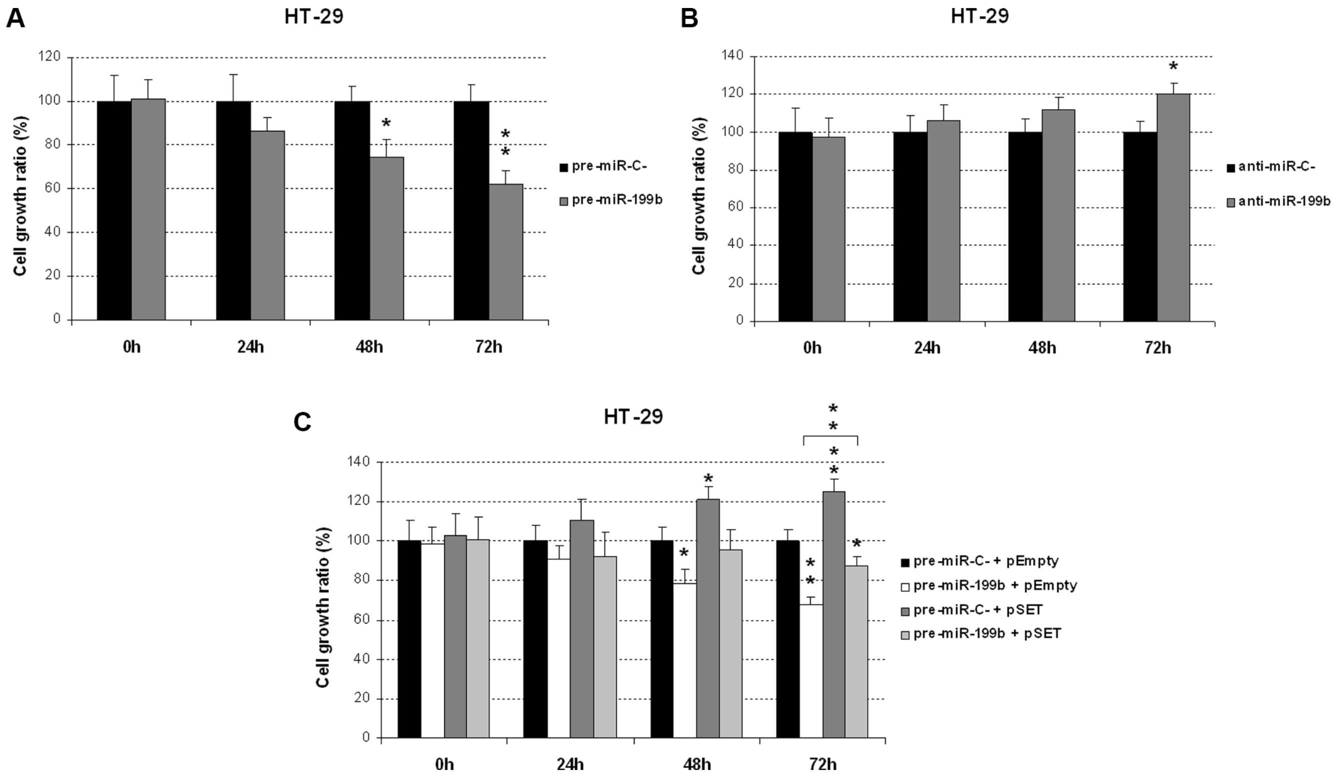

Supplementary Figure S4: MTS assay showing proliferation in HT-29 cells transfected with pre-miR-199b (A), anti-miR-199b (B) or both SET and pre-miR-199b (C); *$P < 0.05$; **$P < 0.01$.

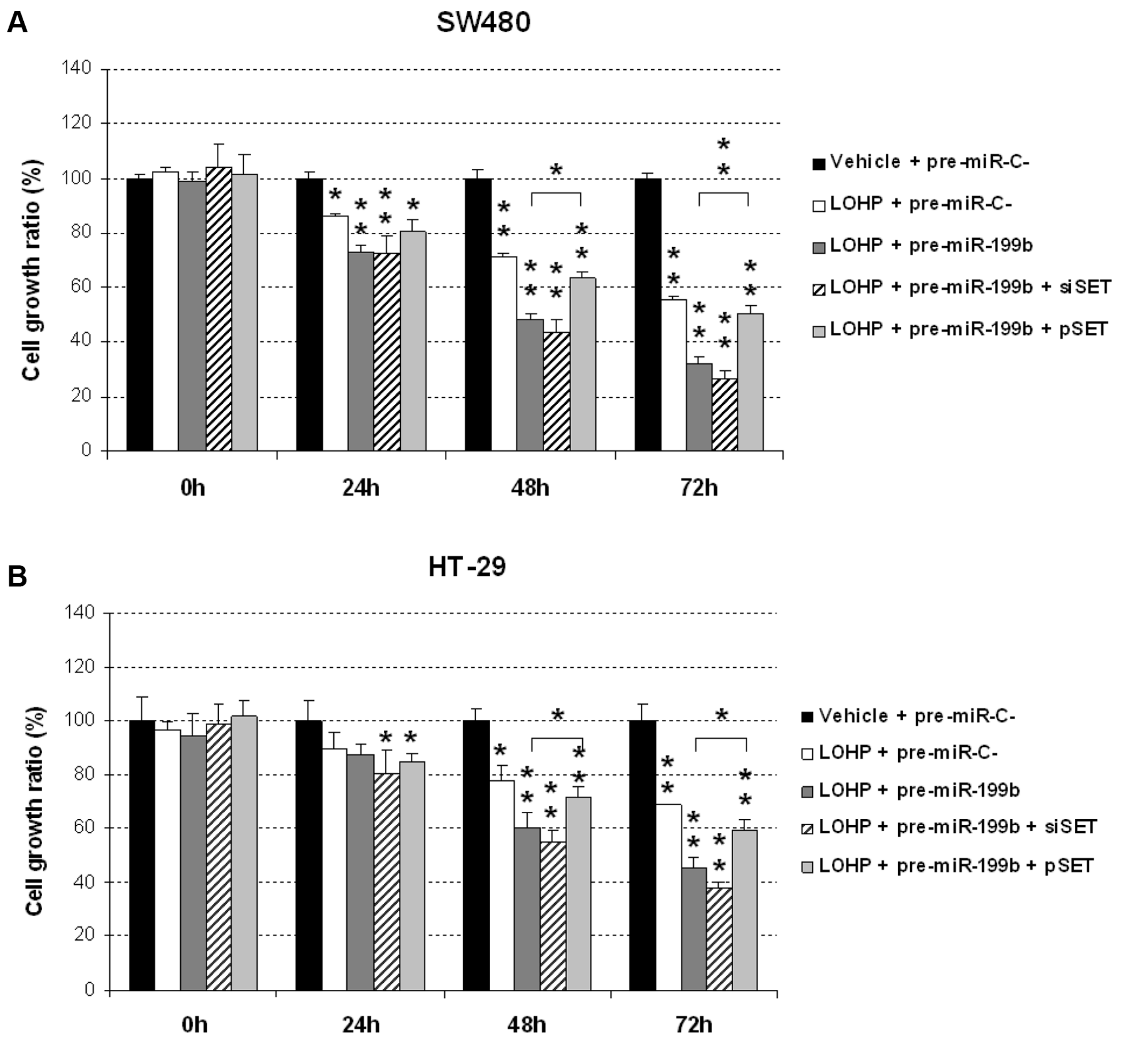

Supplementary Figure S5: MTS assay showing effects of SET modulation in miR-199b-dependent oxaliplain re-sensitation in SW480 (A) and HT-29 cells (B); ****P* **< 0.05; *****P* **< 0.01

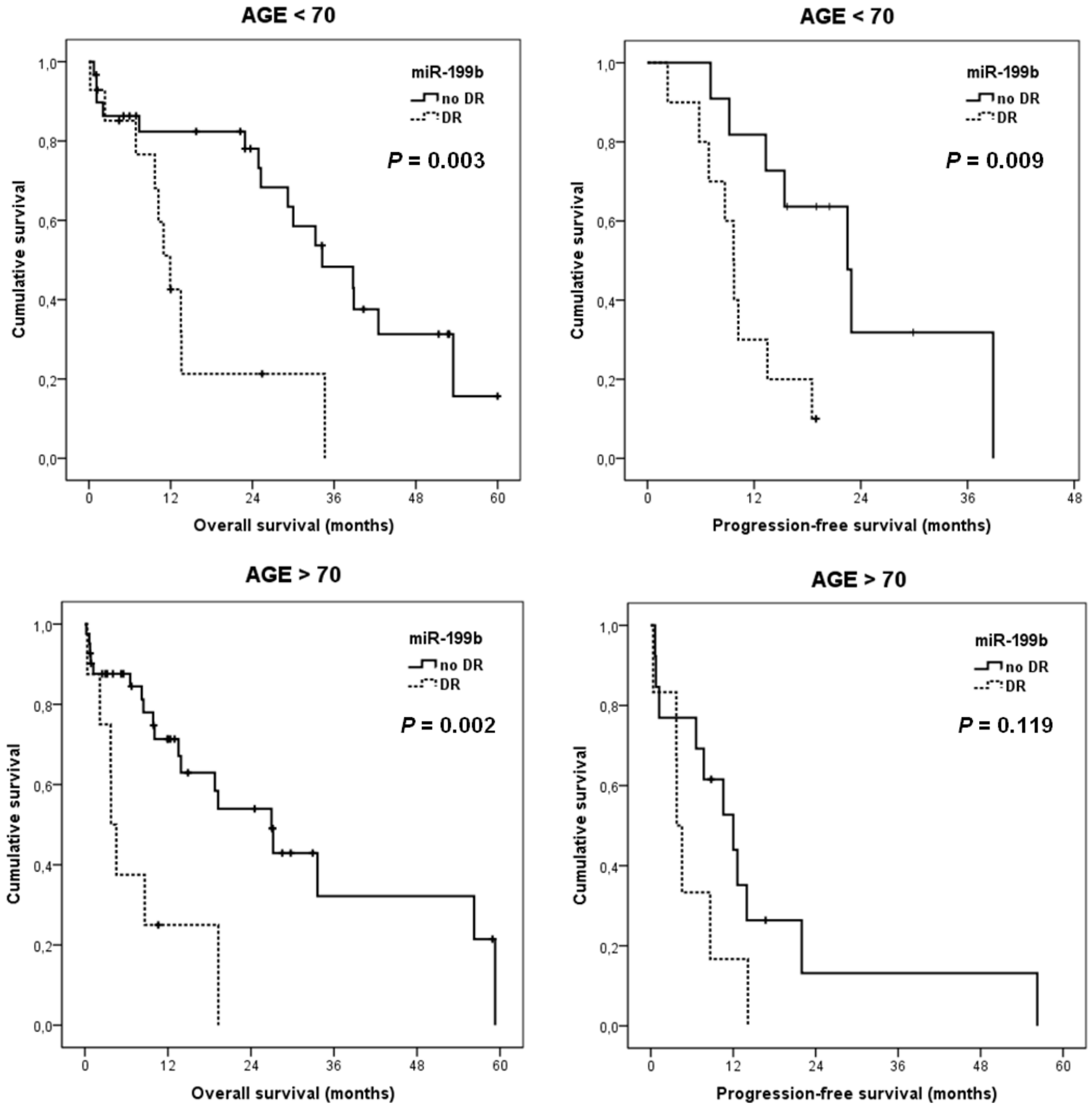

Supplementary Figure S6: Kaplan-Meier analyses of overall and progression-free survival and in the subgroups of patients younger (*N* = 44) and older than 70 years (*N* = 49).

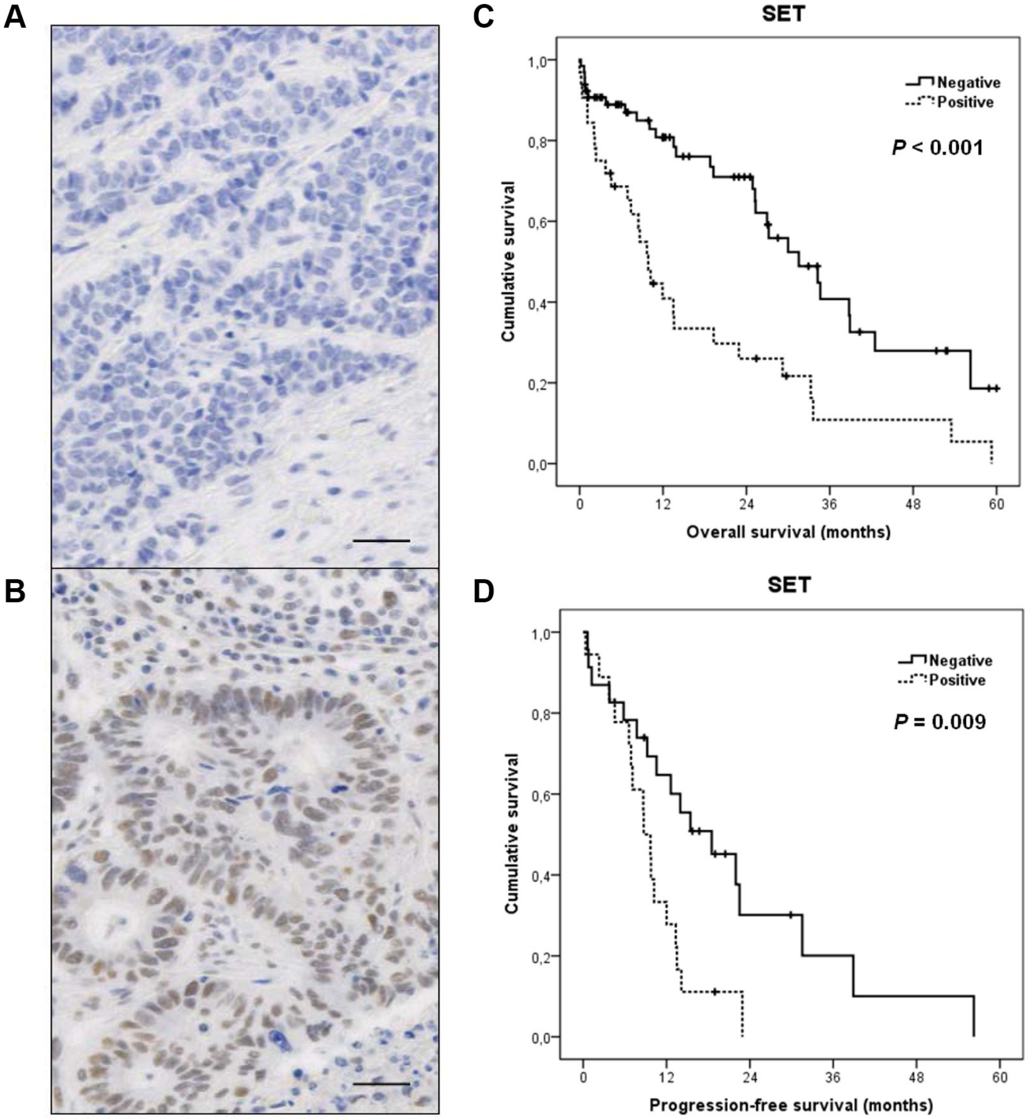

Supplementary Figure S7: Immunohistochemical detection of SET expression in patients with metastatic colorectal cancer. SET negative (**A**) and positive (**B**) staining. The line in A and B shows 25 µm. Magnification x400. Kaplan-Meier analyses for SET in a cohort of 97 patients with metastatic CRC: (**C**) Overall survival; (**D**) Progression-free survival.

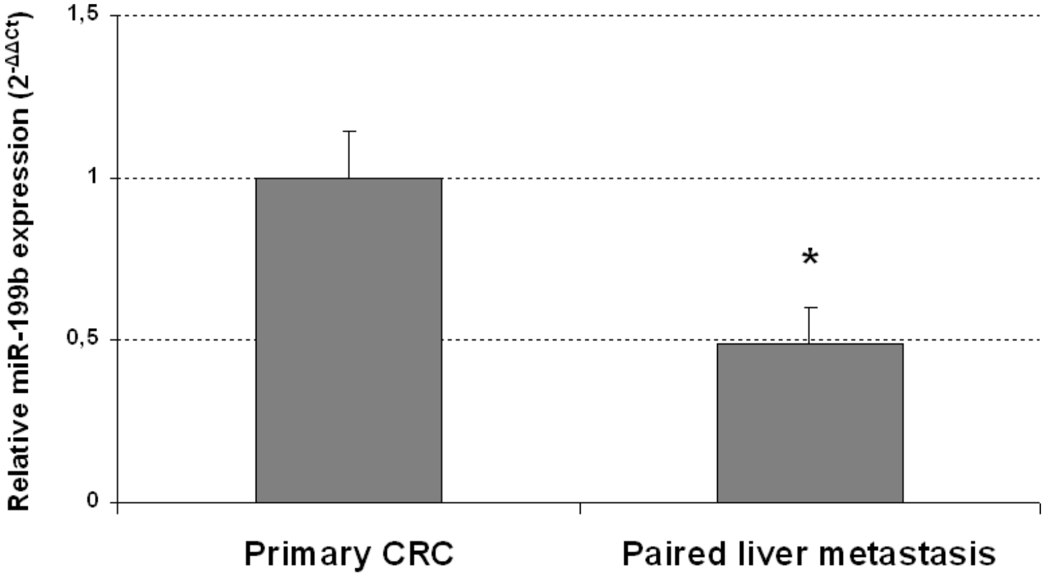

Supplementary Figure S8: Relative miR-199b expression in 10 CRC primary tumors compared with paired liver metastases. A related-samples Wilcoxon signed-ranked test was performed to assess statistical differences. $*P < 0.05$.

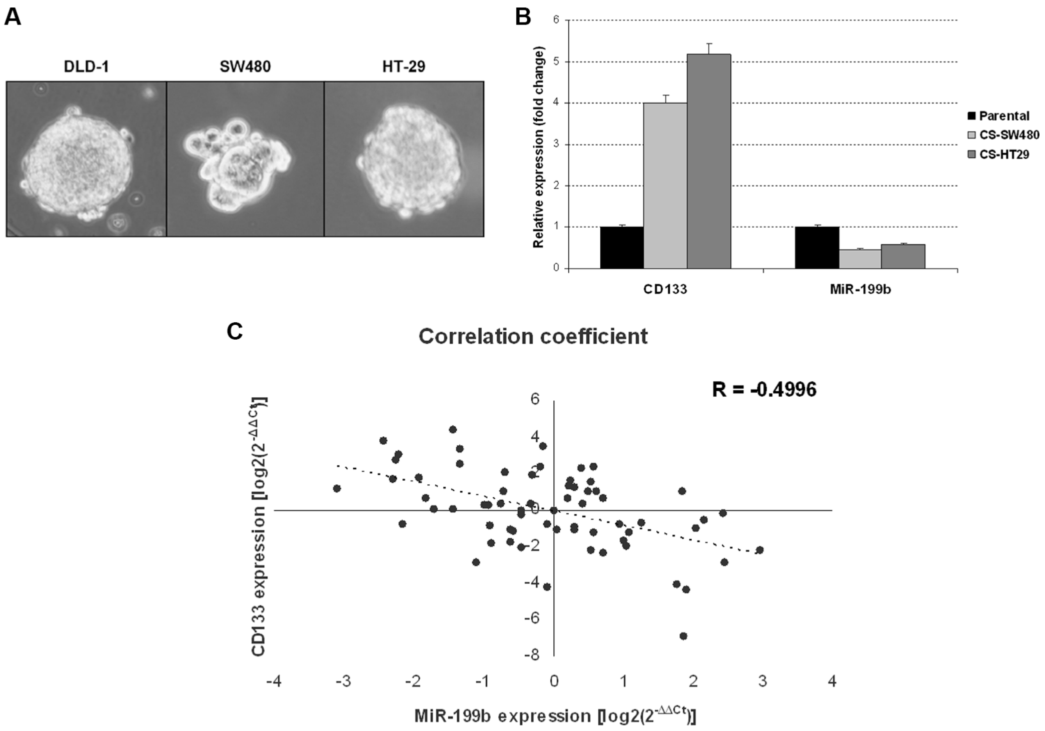

Supplementary Figure S9: (A) Optical microscope images showing DLD-1, SW480 and HT-29-derived colonospheres; (**B**) Quantification of miR-199b and CD133 expression in parental and colonosphere (CS)-derived SW480 and HT-29 cells. We used TaqMan Gene Expression Assays specific for *CD133* (Hs01009259_m1) and GAPDH as internal control; (**C**) Scatter plot showing the correlation between miR-199b and *CD133* expression in 64 metastatic CRC patients.
